# Anxiety and Food Addiction in Men and Women: Results From the Longitudinal LIFE-Adult-Study

**DOI:** 10.3389/fpsyt.2022.914358

**Published:** 2022-06-14

**Authors:** Felix S. Hussenoeder, Alexander Pabst, Ines Conrad, Margrit Löbner, Christoph Engel, Samira Zeynalova, Nigar Reyes, Heide Glaesmer, Andreas Hinz, Veronica Witte, Matthias L. Schroeter, Kerstin Wirkner, Toralf Kirsten, Markus Löffler, Arno Villringer, Steffi G. Riedel-Heller

**Affiliations:** ^1^Institute of Social Medicine, Occupational Health and Public Health, Leipzig University, Leipzig, Germany; ^2^Institute for Medical Informatics, Statistics and Epidemiology, Leipzig University, Leipzig, Germany; ^3^Leipzig Research Centre for Civilization Diseases, Leipzig University, Leipzig, Germany; ^4^Department of Medical Psychology and Medical Sociology, Leipzig University, Leipzig, Germany; ^5^Max Planck Institute for Human Cognitive and Brain Sciences, Leipzig, Germany; ^6^Clinic for Cognitive Neurology, University Hospital Leipzig, Leipzig, Germany; ^7^Department for Medical Data Science, University Hospital Leipzig, Leipzig, Germany

**Keywords:** gender, GAD-7, YFAS, anxiety, longitudinal, food addiction

## Abstract

**Background:**

Anxiety is a widespread phenomenon, and it is connected to disordered eating and obesity. We want to analyze the connection between anxiety and food addiction (FA) over two points in time to better understand the directionality of the association. Since there are gender differences with regard to anxiety and eating, we are also interested in differences between men and women.

**Methods:**

We used data from the population-based LIFE-Adult-Study (*N* = 1,474) at time 1 (baseline) and time 2 (first follow-up) to analyze the connections between anxiety (GAD-7) and FA (YFAS) using a multiple group latent cross-lagged panel model with female and male participants as groups. We controlled for age, marital status, socioeconomic status and social support.

**Results:**

Anxiety (women: β = 0.50, *p* ≤ 0.001; men: β = 0.59, *p* ≤ 0.001) as well as FA (women: β = 0.37, *p* ≤ 0.001; men: β = 0.58, *p* ≤ 0.001) exhibited stability over time for both genders. We found a significant association between anxiety at time 1 and FA at time 2 for women (β = 0.25, *p* ≤ 0.001) but not for men (β = 0.04, *p* = 0.10), and significant associations between FA at time 1 and anxiety at time 2 for women (β = 0.23, *p* ≤ 0.001) as well as men (β = 0.21, *p* ≤ 0.001).

**Conclusion:**

Food addiction longitudinally affects anxiety, independent of gender and other sociodemographic variables. In addition, anxiety affects subsequent FA as well, but only in women. Interventions that address FA could reduce anxiety in men and women, while interventions that mitigate anxiety could help prevent FA in women.

## Introduction

Anxiety and anxiety symptoms are a widespread phenomenon (1, 2). For example, studies report a lifetime prevalence of generalized anxiety disorder (GAD) of around 3.7% (3), and of sub-threshold GAD of around 12.4% (4).

Anxiety is associated with eating-related health outcomes, as research links it to disordered, emotional, uncontrolled, and binge eating behaviors (5–8). In addition, studies show associations with obesity (9, 10) as well as bulimia, binge eating disorder and night eating syndrome that all involve excessive forms of eating behavior and food consumption (8, 11, 12).

While cross-sectional studies suggest an empirical connection between anxiety and eating, the direction of the connection is not clear to date, due to the lack of longitudinal studies in the field. Do increased levels of anxiety contribute to problematic eating behaviors, e.g., as a way of coping, or do problematic forms of eating longitudinally increase anxiety, e.g., *via* disturbing physiological homeostasis? It may also be that both phenomena are mutually dependent on each other in a longitudinal perspective.

In our study, we want to add to the literature by applying a cross-lagged design with two time points in order to evaluate the direction of the effects of the associations between anxiety and food addiction (FA). We chose FA for our analysis since, compared to eating disorders and obesity, it is more common in the general population and more accessible in terms of prevention measures. A current review shows a clear empirical connection between FA and binge eating disorder, bulimia nervosa, and obesity (13). FA has been validated in multiple international studies (14–16), and it is associated with typical addiction phenomena, i.e., brain reward dysfunction, preoccupation, risky use, impaired control, tolerance/withdrawal, social impairment, chronicity, and relapse (17). FA has already been connected to anxiety in cross-sectional research (18–20).

Since the literature shows that women are more likely than men to exhibit anxiety (21, 22) and FA (23, 24) and that the connection between anxiety and disordered eating could be moderated by gender (25, 26), we will further analyze whether the cross-lagged effects between anxiety and FA differ by gender.

## Materials and Methods

### Study Design and Participants

The Adult Study of the Leipzig Research Centre for Civilization Diseases (LIFE) is a population-based cohort study in the city of Leipzig, Germany. It is a collaboration of several clinical and epidemiological research teams, for which 10,000 participants between 18 and 80 years were recruited through age- and gender-stratified random selection by the local residents’ registry office. The only exclusion criterion was being pregnant. The majority of participants (84.9%) were above 40 years of age. The LIFE-Adult baseline examination was carried out between 2011 and 2014, when every participant provided written informed consent prior to participation. Participants underwent a set of assessments, including interviews, questionnaires, and medical examinations. Details on study design, methods and assessments can be found elsewhere (27). The follow up examination took place between 2017 and 2021 with a total of 5,665 individuals completing the postal questionnaires.

For our analyses we included those 1,934 participants that were asked for their eating behaviors and took part in the baseline assessment (time 1) as well as in the follow-up (time 2). We excluded participants who were living in retirement/nursing homes, with relatives or in some form of supported living because we assumed that this would affect their eating behaviors (*N* = 60). In addition, we excluded individuals with diabetes, and those that stated they were treated for a disease, when treatment or disease were likely to have an impact on eating behavior, like ulcer or cancer (*N* = 361). In addition, another 39 individuals had to be excluded due to missing information on covariates, resulting in a final analytical sample size of *N* = 1,474. There was no significant age difference between our sample and the other participants from the LIFE study at baseline, but our sample contained slightly less female participants (53.0% vs. 48.2%).

### Ethics

The LIFE-Adult-Study complies with the ethical standards of the relevant national and institutional committees on human experimentation and with the Helsinki Declaration of 1975, as revised in 2008. The study was approved by the ethics committee of the University of Leipzig.

### Measures

#### Anxiety

In order to measure anxiety, we used the Generalized Anxiety Disorder Scale-7 (GAD-7), (28, 29) which contains seven items that can be answered on a scale from “0” (=never) to “3” (=almost every day). The items refer to typical anxiety symptoms, like worrying, nervousness, and irritability, and higher scores represent higher levels of anxiety.

#### Food Addiction

We used the Yale Food Addiction Scale (YFAS); (30, 31) to assess FA. The scale contains 25 items with mixed response categories (dichotomous and Likert-type). The seven subscales of the YFAS represent the criteria for an eating addiction in line with the guidelines for substance dependence according to DSM-IV, like control over consumption and withdrawal. They were computed using the algorithm proposed in Gearhardt et al. (30). The eighth item (clinical significance) was excluded, so that the YFAS measurement resembles a symptom count without diagnosis at both times. Scores range from 0 to 7 with higher scores representing higher levels of FA.

#### Sociodemographic Variables and Covariates

Participants were asked for information on age, gender, marital status, and medical history in standardized interviews by trained study personnel. They also provided information on education, equivalent household income, and occupational status that was used to compute socioeconomic status (low, medium, and high); (32). We assessed social support *via* the 5-item ENRICHD Social Support Scale (33). We decided to include social support as a covariate based on our own theoretical considerations as well as on the literature (34).

### Statistical Analyses

Descriptive statistics of the analytical sample were estimated using Stata version 16 SE (Stata Corp., College Station, TX, United States). In particular, gender-stratified means with SDs and numbers of cases with percentages were reported for quantitative and qualitative measures, respectively.

**FIGURE 1:**
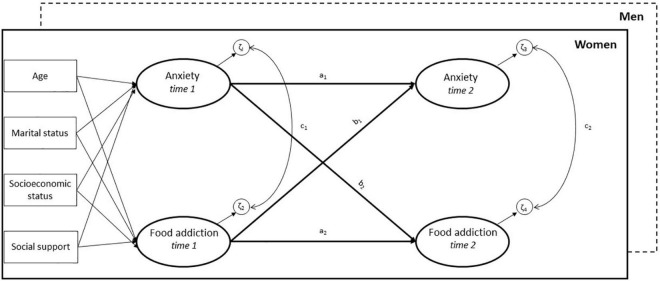
Multiple-group latent autoregressive cross-lagged panel model of the association between anxiety and food addiction. a_1_, a_2_: autoregressive paths, b_1_, b_2_^:^ cross-lagged paths; c_1_, c_2_^:^ cross-sectional paths. The measurement models for the latent variables with pairwise correlated errors over time are not shown. Model adjusted for age, marital status, socioeconomic status and social support at time 1.

The bidirectional relationships between FA and anxiety were examined using a latent autoregressive cross-lagged panel model with multiple groups, estimated in Mplus 8.6 (35). The model consists of three parts: the autoregressive paths a_1_ and a_2_ indicate the intraindividual stability of FA and anxiety over time (Figure 1). The two cross-lagged paths b_1_ and b_2_ represent the reciprocal effect of FA at time 1 on anxiety at time 2 and vice versa. Finally, the cross-sectional paths c_1_ and c_2_ model the covariance between FA and anxiety within each wave of assessment. The multiple-group option in Mplus allows estimating and comparing the depicted cross-lagged path model between men and women simultaneously.

Individual items of the GAD-7 and the subscale scores of the YFAS were entered as ordered categorical in Mplus and the constructs were modeled as latent variables. First, both constructs were evaluated separately using confirmatory factor analysis (CFA). With regard to the YFAS, the subscale “attempts” did not significantly predict the latent variable at time 1 (β = 0.197, *p* = 0.056) and time 2 (β = 0.010, *p* = 0.937) and was subsequently excluded from the analyses. Second, measurement invariance across time (i.e., time 1 and time 2) and across groups (i.e., men and women) was evaluated by introducing equality constraints on model parameters (e.g., factor loadings, intercepts, and variances) in a series of models with increasingly restrictive hypotheses. Parameters that proved not invariant, as indicated by model fit indices and chi-square difference tests in Mplus, were allowed to vary across time and groups. Error terms of the GAD-7 items and the YFAS subscales were set to be pairwise correlated over time, and factor means of the latent variables were allowed to vary by gender. Next, the partially invariant measurement models for FA and anxiety were combined to estimate the cross-lagged panel model shown in Figure 1. The model was finally adjusted for age, marital status, SES and social support at time 1. Results are presented as fully standardized (STDYX) regression coefficients with 95% confidence intervals for the paths a_1_ to c_2_ in the final cross-lagged model.

Since items of the GAD-7 and the computed subscale scores of the YFAS were ordered categorical, the WLSMV estimator in Mplus was used for the estimation of effects. Missingness on single indicators of the GAD-7 and the YFAS were handled using Full Information Maximum Likelihood (FIML) estimation, as implemented in Mplus. As indices of goodness-of-fit, the Tucker-Lewis fit index (TLI), the comparative fit index (CLI) and the root mean square error of approximation (RSMEA) were computed, with values below 0.06 for the RMSEA, and values above 0.95 for the TLI and CFI indicating a good model fit (36). All tests were two-tailed with *p* < 0.05 indicating statistical significance.

## Results

Our sample included 711 (48.2%) female and 763 (51.8%) male participants with an average age of 57.6 (female) and 58.4 (male) years. Table 1 gives an overview of the general characteristics of our sample.

**TABLE 1:** General characteristics of the study population.

	Women (*N* = 711)	Men (*N* = 763)	Total (*N* = 1,474)
Age *n.s.*	57.6 (14.5)	58.4 (14.9)	58.0 (14.7)
Marital status***			
Married	431 (60.6%)	515 (67.5%)	946 (64.2%)
Single	148 (20.8%)	160 (21.0%)	308 (20.9%)
Divorced	80 (11.3%)	65 (8.5%)	145 (9.8%)
Widowed	52 (7.3%)	23 (3.0%)	75 (5.1%)
Socioeconomic status^1^**			
Low	119 (16.7%)	100 (13.1%)	219 (14.9%)
Medium	436 (61.3%)	443 (58.1%)	879 (59.6%)
High	156 (21.9%)	220 (28.8%)	376 (25.5%)
Social Support (ESSI; 5 – 25) *n.s.*	22.5 (3.2)	22.3 (3.4)	22.4 (3.3)
**Anxiety (GAD-7; 0 – 21)**			
Time 1***	3.5 (3.2)	2.5 (2.5)	3.0 (2.9)
Time 2***	3.7 (3.5)	2.7 (2.8)	3.2 (3.2)
**Food addiction (YFAS; 0 – 7)**			
Time 1 *n.s.*	1.4 (0.9)	1.3 (0.7)	1.3 (0.8)
Time 2*	1.4 (0.9)	1.3 (0.7)	1.3 (0.8)

*p ≤ 0.05; **p ≤ 0.01; ***p ≤ 0.001; n.s., not significant (referring to differences between female and male participants). Continuous variables are given as mean (standard deviation), and p-values refer to independent t-tests; categorical variables are displayed as numbers (percentages), and p-values refer to Chi 2 –tests.

*^1^Socioeconomic status was computed based on education, occupational status, and equivalent household income (32).*

Table 2 depicts the correlations between key variables of our analysis, and shows that all of them are significantly correlated.

**TABLE 2:** Correlations of key variables for women and men.

	1	2	3	4
**Women**				
1. Anxiety time 1	–			
2. Anxiety time 2	0.52***	–		
3. Food addiction time 1	0.23***	0.27***	–	
4. Food addiction time 2	0.24***	0.20***	0.28***	–
**Men**				
1. Anxiety time 1	–			
2. Anxiety time 2	0.52***	–		
3. Food addiction time 1	0.16***	0.16***	–	
4. Food addiction time 2	0.09*	0.10*	0.23***	–
**Total**				
1. Anxiety time 1	–			
2. Anxiety time 2	0.53***	–		
3. Food addiction time 1	0.21***	0.23***	–	
4. Food addiction time 2	0.19***	0.17***	0.26***	–

Pearson’s correlation, two-tailed. *p ≤ 0.05; **p ≤ 0.01; ***p ≤ 0.001.

### Model Fit

**FIGURE 2:**
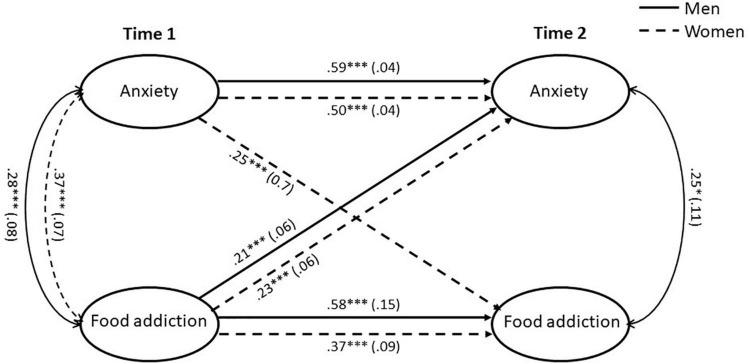
Multiple-group latent autoregressive cross-lagged panel model with standardized beta coefficients and standard errors in parentheses. The model only displays the significant paths for men and women. The effects of control variables (age, marital status, socioeconomic status, social support) on anxiety and food addiction at time 1 were included in the estimation but not shown for ease of presentation. Sample size: 1,474 (48.2% female). *p ≤ 0.05; ^***^p ≤ 0.001.

After adjustment for covariates, the final multiple-group cross-lagged panel model with partial measurement invariance yielded an excellent fit of the data (χ^2^(988) = 1,372.07, *p* > 0.001; CFI = 0.971; TLI = 0.970; RMSEA = 0.023). This model with gender-specific paths for the autoregressive, cross-sectional and cross-lagged effects fitted the data statistically better than a model with each of the effects constrained across gender (χ^2^(4) = 17.33, *p* = 0.002). Standardized coefficients and standard errors of the significant paths obtained from the final model are shown for both genders in Figure 2.

### Autoregressive Paths

Autoregressive paths (a_1_, a_2_) represent the stability of a concept over time. Both autoregressive effects for anxiety (women: β = 0.50, *p* ≤ 0.001; men: β = 0.59, *p* ≤ 0.001) as well as for FA (women: β = 0.37, *p* ≤ 0.001; men: β = 0.58, *p* ≤ 0.001) were statistically significant for both genders.

### Cross-Sectional Paths

Cross-sectional paths (c_1_, c_2_) represent correlations between concepts at either time 1 or time 2. Female participants showed a significant correlation between anxiety and FA at time 1 (*r* = 0.37, *p* ≤ 0.001) but no significant correlation at time 2 (*r* = −0.02, *p* = 0.78), while male participants exhibited significant correlations at time 1 (*r* = 0.28, *p* ≤ 0.001) and time 2 (*r* = 0.25, *p* ≤ 0.05).

### Cross-Lagged Paths

Cross-lagged paths (b_1_, b_2_) represent prospective bidirectional associations between one concept and the other over the two points of time. We found an association between anxiety at time 1 and FA at time 2 for women (β = 0.25, *p* ≤ 0.001) but not for men (β = 0.04, *p* = 0.69). In addition, there were significant associations between FA at time 1 and anxiety at time 2 for women (β = 0.23, *p* ≤ 0.001) as well as men (β = 0.21, *p* ≤ 0.001).

## Discussion

Our study addressed the associations between anxiety and FA in a longitudinal design, showing the significant stability of both constructs over time. There was a significant effect of FA at time 1 on anxiety at time 2 for both genders. Vice versa, only women showed a significant effect of anxiety at time 1 on FA at time 2.

The stability of anxiety (37, 38) and FA (39, 40) over time that we have obtained from our data is also reflected in the literature. In addition, the higher levels of anxiety in women at both times of measurement resonate with other international studies (41, 42). With regard to FA, we found no gender difference at time 1 but a significantly yet only slightly higher score for women at time 2. These results are in line with the literature that suggest either no gender effects (43, 44) or higher values for women (24, 45).

We found a cross-sectional association between anxiety and FA for women as well as men at time 1 and for men at time 2, which matches with other studies that suggest associations between FA and a higher prevalence of anxiety disorders in obese patients seeking bariatric surgery (46) and between FA and anxiety in general (19). The results indicate that both concepts are interrelated, and the lack of a significant association for women at time 2 that is not reflected in the correlational analysis (Table 2) can be seen as a consequence of the inclusion of control variables.

We also found evidence for cross-lagged effects. There is a significant effect of anxiety at time 1 on FA at time 2 for women, indicating that anxiety has different implications depending on gender. This gender-specific effect could be explained by rumination, a cognitive process and maladaptive strategy for emotion regulation that involves repetitive thoughts about negative experiences and emotions. Rumination is empirically associated with both, anxiety and pathological forms of eating (47–49), and it has been connected to a variety of addictive behaviors, e.g., related to alcohol, work, or social media use (50–52). Furthermore, a current study suggests that targeting rumination could be important for reducing disinhibited eating patterns in women with normal body weight (53). Since women are more likely to ruminate than men (54), when men and women experience the same level of anxiety, women will be much more affected by rumination that then contributes to FA. This interpretation is in line with research that suggests that rumination mediated the connection between gender and food craving, binge eating, and eating pathology (55), that rumination can increase problematic alcohol and substance abuse, especially in women (56), and that women are more likely to exhibit emotional eating as a reaction to negative emotions (5, 57). Rumination has repeatedly been associated with exacerbating and maintaining psychopathology and physiological stress responses, prolonging negative emotional states, increasing negative emotional reactivity, interfering with problem solving, and acting as a transdiagnostic mental vulnerability (56). In that way, rumination that is associated with anxiety at time 1 could, in addition to maintaining anxiety over time as it is reflected in significant and substantial autocorrelations, set the stage for FA at time 2. On an applied level, our results indicate that interventions that mitigate anxiety could help prevent FA in women, who are more often affected by anxiety than men (58, 59). Meta-analyses show that measures based on cognitive behavioral therapy, delivered both online and offline (60, 61), are effective against anxiety, and a current study suggests that irrational beliefs could be a source of anxiety and a potential target for treatment in FA (62). By addressing anxiety, mental health professionals could not only mitigate FA in women, but they could also reduce the likelihood of a variety of negative health behaviors and outcomes that are related to FA, from unhealthy lifestyle habits (63) to eating disorders, mental illnesses, and obesity (19, 45, 64). Overweight and obesity are major risk factors for a variety of disorders, and they bear enormous costs for societies worldwide (65).

Our results further indicate a cross-lagged effect of FA at time 1 on anxiety at time 2 in both men and women. This could be a consequence of the fact that the overconsumption of food that is a central element of FA alters brain functioning and physiology, which then affects anxiety. Accordingly, there is a plethora of neurobiological studies that link the consumption of food that is high in calories, sugar, or fat to anxiety-like behaviors in rats and that emphasize the roles of certain brain circuits, neurobiological processes, and the immune system (66–68). In addition, research with human participants links disordered eating to subsequent increased anxiety and anxiety disorders (69, 70). Hence, interventions that address FA behaviors and food consumption could mitigate anxiety in men and women. Studies that address overeating and binge eating behaviors suggest that cognitive interventions that address internal food-related biases and response inhibition training, mindfulness-based interventions, and increasing physical activity could be promising avenues to address FA (71–73). A current study suggests that the treatment of FA could also benefit weight-related self-stigma and binge eating (74).

### Limitations

While this study has several advantages, e.g., the large dataset and a longitudinal design, there are also certain limitations. First, FA was assessed *via* self-report, therefore we cannot rule out that there is a certain bias. Second, while we included established control variables in our model, there could be other variables that affect both anxiety and FA, e.g., specific personality traits, and future research may benefit from including them.

## Conclusion

Our results show that FA longitudinally affected anxiety in both men and women, and that anxiety affected subsequent FA only in women. Hence, interventions that address FA could reduce anxiety in both genders, while interventions that mitigate anxiety could help prevent FA in women.

## Data Availability Statement

The data analyzed in this study is subject to the following licenses/restrictions: The data that support the findings of this study are available from the corresponding author upon reasonable request. Requests to access these datasets should be directed to FH, Felix.Hussenoeder@medizin.uni-leipzig.de.

## Ethics Statement

The LIFE-Adult-Study complies with the ethical standards of the relevant national and institutional committees on human experimentation and with the Helsinki Declaration of 1975, as revised in 2008. The study was approved by the Ethics Committee of the University of Leipzig. The patients/participants provided their written informed consent to participate in this study.

## Author Contributions

FH, AP, IC, and SR-H designed the study. FH and AP conducted the statistical analysis and literature searches. FH wrote the first draft of the manuscript. MGL, CE, SZ, NR, HG, AH, VW, MS, KW, TK, MKL, and AV contributed to the data and/or expertise. All authors contributed to and have approved the final manuscript.

## Conflict of Interest

The authors declare that the research was conducted in the absence of any commercial or financial relationships that could be construed as a potential conflict of interest.

## Publisher’s Note

All claims expressed in this article are solely those of the authors and do not necessarily represent those of their affiliated organizations, or those of the publisher, the editors and the reviewers. Any product that may be evaluated in this article, or claim that may be made by its manufacturer, is not guaranteed or endorsed by the publisher.
